# Familiarity with a Tool Influences Peripersonal Space and Primary Motor Cortex Excitability of Muscles Involved in Haptic Contact

**DOI:** 10.1093/texcom/tgaa065

**Published:** 2020-09-15

**Authors:** M Biggio, A Bisio, L Avanzino, P Ruggeri, M Bove

**Affiliations:** Department of Experimental Medicine, Section of Human Physiology and Centro Polifunzionale Scienze Motorie, University of Genoa, 16132 Genoa, Italy; Department of Experimental Medicine, Section of Human Physiology and Centro Polifunzionale Scienze Motorie, University of Genoa, 16132 Genoa, Italy; Department of Experimental Medicine, Section of Human Physiology and Centro Polifunzionale Scienze Motorie, University of Genoa, 16132 Genoa, Italy; Ospedale Policlinico San Martino, IRCSS, 16132, Genova, Italy; Department of Experimental Medicine, Section of Human Physiology and Centro Polifunzionale Scienze Motorie, University of Genoa, 16132 Genoa, Italy; Department of Experimental Medicine, Section of Human Physiology and Centro Polifunzionale Scienze Motorie, University of Genoa, 16132 Genoa, Italy; Ospedale Policlinico San Martino, IRCSS, 16132, Genova, Italy

**Keywords:** motor imagery, multisensory integration, peripersonal space, tool-use, transcranial magnetic stimulation

## Abstract

Long-term experience with a tool stably enlarges peripersonal space (PPS). Also, gained experience with a tool modulates internal models of action. The aim of this work was to understand whether the familiarity with a tool influences both PPS and motor representation. Toward this goal, we tested in 13 expert fencers through a multisensory integration paradigm the embodiment in their PPS of a personal (pE) or a common (cE) épée. Then, we evaluated the primary motor cortex excitability of proximal (ECR) and distal (APB) muscles during a motor imagery (MI) task of an athletic gesture when athletes handled these tools. Results showed that pE enlarges subjects’ PPS, while cE does not. Moreover, during MI, handling tools increased cortical excitability of ECR muscle. Notably, APB’s cortical excitability during MI only increased with pE as a function of its embodiment in PPS. These findings indicate that the familiarity with a tool specifically enlarges PPS and modulates the cortical motor representation of those muscles involved in the haptic contact with it.

## Introduction

Human daily life is characterized by the use of various instruments that modify the way we interact with the external world. People become skilled in handling tools they often use and, sometimes, these tools can nearly become an extension of their body. In sports, players perform the same athletic gestures several times per day for many years in order to improve their performance. In many sports, athletes have their own tool, whose handling is a fundamental part of the action. It is known that the representation of a body part can be modified by using a tool that mimics its morpho-functional characteristics (Cardinali et al. 2016). Coelho et al. (2019) demonstrated that in a group of expert baseball players the hand representation is stably reduced with respect to a group of players who were trained with a baseball glove for a short amount of time.

Further, a tool modifies our field of action, thereby changing our efficacy in surrounding space. Experimental evidence indicates that short- and long-term familiarity of limbs with specific objects shapes both spatial and bodily representations (Cardinali, Brozzoli et al. 2009), such as the integration of tools into the human body schema (Aglioti et al. 1996; Berlucchi and Aglioti 1997; Pegna et al. 2001; Maravita and Iriki 2004) and the extension of the peripersonal space (PPS) (Farnè and Làdavas 2000; Holmes 2012; Canzoneri, Ubaldi et al. 2013). Both constructs share multisensory and motor properties, since they are modulated by action-dependent manipulations (Angelo et al. 2018; Schettler et al. 2019). PPS represents the area directly surrounding the body (Rizzolatti et al. 1997) in which we can directly act, characterized by a high degree of multisensory integration (Graziano 1999; Farnè and Làdavas 2000; Cardinali, Brozzoli et al. 2009; di Pellegrino and Làdavas 2014). It is also considered a multisensory-motor interface that led to locate sensory stimuli in the external space in order to generate a goal-oriented behavior when possible in the reaching distance (di Pellegrino and Làdavas 2014; de Vignemont and Iannetti 2015; Angelo et al. 2018; Serino 2019).

In a previous study, we showed that the extension of tennis players’ PPS is differently modulated by the familiarity with the tennis racket with respect to novices (Biggio et al. 2017). Subjects performed a multisensory integration paradigm, reacting as fast as possible to tactile stimuli, ignoring a concurrent sound originating either near the subjects’ hand or near a handled tool, but far from the body. Similar reaction times to stimuli associated with the near and far sounds indicate the integration of the handled tool inside the PPS (Serino et al. 2007). We found that athletes’ long-term experience with a tool led to a permanent enlargement of the boundaries of their PPS representations for the specific tennis racket used during their daily sport activity. On the contrary, with an unfamiliar generic tennis racket, athletes and novices showed a comparable pattern (Biggio et al. 2017). In expert athletes the long-term physical training with sport-related tools leads to functional and structural changes in multiple brain areas (Jafarzadehpur and Yarigholi 2004; Fourkas et al. 2008; Ohguni et al. 2009; Di et al. 2012), and it is also reflected in behavioral measures, such as duration of imagined movements (Bisio et al. 2014). Coherently, Wang et al. (2014) showed an increased cortical excitability in badminton players when they imagined running a sportive gesture while holding the badminton racket but not a plastic bar. Motor imagery (MI) is a mental process that consists of imagining a motor task in the absence of movement and muscular activity (Hanakawa et al. 2003). MI and executed movement are similar in many aspects; in fact, their neuronal activity overlap, as shown by neuroimaging and neurophysiological studies (Decety and Jeannerod 1995; Rossini 1999; Facchini et al. 2002; Munzert and Zentgraf 2009). The development of internal models of action is a requirement for motor learning and for the generation of skilled actions, since the dynamics of our body may change in different situations, as during the use of tool, which have their own intrinsic dynamics. Thus, we need to update or create new models of action through experience (Rosenbaum et al. 1993; Wolpert 1997; Wolpert and Flanagan 2001). Since experience is based on previous individual circumstances, haptic information received from proprioceptors and mechanoreceptors located in the palm of the hand about object weight and material are integrated in the brain to generate perceptual estimate of a given tool in action (van Polanen et al. 2019). Practice of actions with tools results in stored representations that integrate information about forces and resistance, haptic and visual kinematic information, and properties of the object and the environment (White 2012).

Tool integration in internal models can be therefore investigated in athletes through MI. Fourkas et al. (2008) showed that cortical excitability in athletes increased when they imagined to execute a gesture related to their sport rather than related to another. Recent studies analyzed the role of the tools, by comparing the effects evoked by the specific object for a sport and an unrelated equipment by means of behavioral tasks and neurophysiological methods. Bisio et al. (2014) found that, in tennis players, handling a racket rather than an umbrella during MI induced a better isochrony between real and imagined sport-related movements. These results indicate that a long-term training with a sport tool drives the integration of such tool in athletes’ motor plans. This suggests that learning actions involving tool-use is a multisensory process in which the haptic contact between the body and the tool plays an important role, since stored representations carry information regarding forces based on prior haptic experience (White 2012). Interestingly, previous studies found divergent results when exploring distal and proximal muscles’ cortical excitability (Fourkas et al. 2008; Wang et al. 2014).

However, whether this modification in space perception is related to an integration of the personal sport-tool in the cortical motor representation is yet to be demonstrated.

The aim of this work was to explore a possible integration of a long-term embodied tool in the cortical motor representation. To do that, we investigated whether a long-term use of a tool during sport activity can induce its embodiment in elite athletes and how this condition can influence the excitability of the primary motor cortex during MI. Embodiment of tools is a multisensory integration process representing the sensation that those objects become part of our body, in the same way limbs belong to us. It includes the sense of ownership, the sense of agency, and the sense of self-location over the embodied object (Kilteni et al. 2012; Schettler et al. 2019).

Also, since cortical excitability during MI has been proven to be selective for the muscle directly involved in the imagined action (Fadiga et al. 1999; Hashimoto and Rothwell 1999; Guillot et al. 2007), we hypothesize that the muscle activation could change causing the recall of different internal models. We suggest that expert fencers could have enough experience to use an épée but not enough experience with that specific object to anticipate the specific sensation of the hand.

Since we believe that haptic contact might play an important role in tool embodiment, we investigated whether the excitability of proximal and distal upper limb muscles could be differently modulated when handling a tool integrated in the PPS.

Therefore, we tested 13 elite fencers, comparing the embodiment of their personal épées with a common 1 through a multisensory integration paradigm. Then, we investigated by means of transcranial magnetic stimulation (TMS) the integration of these tools in motor plans, testing the cortical excitability of abductor pollicis brevis (APB) and extensor carpi radialis (ECR) muscles during MI of a fencing-gesture while subject held the personal and common épée.

## Material and Methods

### Participants

A total of 13 expert fencers (7 males and 6 females, mean ± SE = 25.77 ± 2.67 years) took part in the experiment. Only subjects specialized in épée, 1 of the 3 weapons used in fencing, were selected. Agonist athletes of national and international level composed the group. Subjects underwent a questionnaire to explore their athletic career. The years of experience ranged from 10 to 33 (mean ± SE = 14.97 ± 1.64) and the hours of weekly practice ranged from 5 to 28 (mean ± SE = 11.44 ± 1.61). All subjects participated in international competitions. Two of them started their careers with a different weapon (foil or saber), but only used the épée sword for most of their sports career.

All the participants were right-handed, as determined by the Edinburgh Handedness Inventory (Oldfield 1971), had normal hearing and touch, and had no contraindication to TMS. The study was conducted in accordance with the Declaration of Helsinki, and approved by the local ethics committee. Each participant signed an informed consent prior to the study.

### Procedure

Subjects underwent 2 experimental sessions, in 2 different days. Both experimental sessions involved 2 objects: a common épée (cE), the same sword given to all of subjects, and the personal épée (pE), the tool each fencer uses during her/his sport activity. Firstly, we tested whether the personal épée was integrated into fencers’ PPS better than a common épée, employing a multisensory integration paradigm (Serino et al. 2007; Biggio et al. 2017). Then, we investigated if the tool was included in the internal motor representation, testing the cortical excitability during an MI task while subjects held 1 of the 2 épée.

### Multisensory Integration Experiment

Subjects sat on a chair with the back of the right hand always lying on a table. They performed a simple detection task during which they were required to verbally respond saying “tah!” as soon as they perceived an electrical tactile stimulus. The tactile stimulus was administered at the right wrist using a surface bipolar electrode attached with a velcro strap and connected to a Digitimer constant current stimulator (DS7AH HV, Digitimer Ltd, UK). Stimulus intensity was set at the sensory threshold of each subject. Participants’ verbal responses were acquired through a microphone positioned around the neck.

A task-irrelevant sound (a 150-ms burst of pink noise), which subjects were instructed to ignore, was presented simultaneously to the electric stimulus. The sound was originated from 1 of the 2 identical loudspeakers that were placed 1 in close proximity to the right hand, at about 30 cm from the body (near position), and the other at a distance of about 110 cm from the other 1 (far position). The volume of the speakers was singularly regulated so that the intensity of the near and far sound was equal (70 dB) as measured by a sound meter at subjects’ right ear. The tactile and the acoustic stimuli originating from the loudspeaker near the hand were delivered simultaneously. The far sound started about 3 ms before the onset of the tactile stimulus, in order to compensate for the delayed arrival of the sound, due to the spatial distance. A custom-made MatLab® software managed the synchronization between the electrical and audio stimuli and the order of the trials.

### Experimental Design

**Figure 1:**
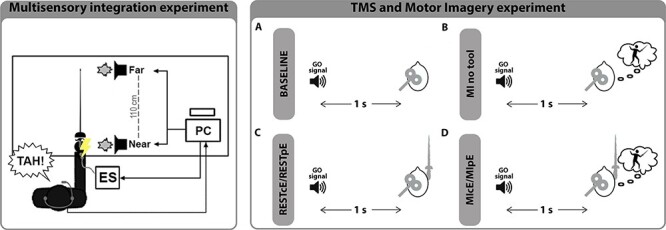
Experimental protocols. Multisensory integration experiment. Participants sat on a chair with the back of the right hand lying on a table. They were requested to verbally respond (saying “TAH!”) to an electrical tactile stimulus administered from the electrical stimulator (ES) in correspondence of the right wrist. Participants’ verbal responses were acquired through a microphone positioned around the neck. Simultaneously to the electric stimulus, a task-irrelevant sound was presented either in close proximity to the right hand (Near) or at a distance of about 110 cm (Far). A personal computer (PC) controlled the order and the synchronization of the stimuli. Figure refers both to the set up in the common épée (cE) session and in personal épée (pE). TMS and Motor Imagery experiment: Panel A represents BASELINE session. Subjects seated with their eyes closed without any tool and no mental task was required. Panel B represents “MI no-tool” session. Subjects seated with their eyes closed without any tool. They had to imagine a parry IV-attack combo after the GO signal. TMS was delivered 1 s after the sound, during the MI. Panel C represents REST sessions. Subjects had to held common épée (cE) or personal épée (pE) without any mental task. TMS was delivered 1 s after the GO sound. Panels D represents different trials of “MI tools” session. Subjects had to held common épée (cE) or personal épée (pE) and were instructed to imagine a parry IV-attack combo. TMS was delivered during MI.

The multisensory integration paradigm was repeated in 2 sessions, whose order was counterbalanced among the subjects. Participants were blindfolded, sitting at a table handling the épée in the right hand. The hand lied with the back on the table, close to the near loudspeaker. They had to answer verbally, as soon as possible, to the tactile stimulus, ignoring the nontarget auditory stimulus. In the common épée (cE) session, subjects underwent the multisensory integration paradigm holding with the right hand a 110 cm long épée that weighed 750 g (the same object for everyone) at the level of the handle, which was settled in correspondence of the near loudspeaker. The remainder of the tool lied on the table so that the tip of the sword was placed in correspondence to the far loudspeaker. In personal épée (pE) session, participants performed the multisensory integration paradigm holding the sword they regularly used to train themselves (Fig. 1—multisensory integration experiment). The personal épées were all long 110 cm and weighed 750 g. Between sessions, subjects had the possibility to lift and settle the swords for the following session, but they remained blindfolded.

Each session consisted of 3 conditions randomly executed: 30 trials during which a tactile stimulus was coupled with the near sound (Near condition), 30 trials where the tactile stimulus was coupled with the far sound (Far condition), and 30 catch trials (control condition) where subjects only heard either the Near (15) or the Far (15) sounds and they had to prevent themselves from answering. Catch trials were performed in order to avoid habituation. A familiarization phase, consisting of 3 repetitions of each experimental condition, including the catch trials, preceded the beginning of the experiment.

### Data Processing and Statistical Analysis

A custom-made MatLab software was used to analyze the audio traces of the subjects’ verbal answers. From each trace, the reaction time (RT, ms) was calculated as the time elapsed between the onset of participant’s verbal response and the delivery of the tactile stimulus in both Far (RT_Far_) and Near (RT_Near_) conditions. Responses higher or lower than 2 standard deviations from the individual mean RT value were treated as outliers and were removed from the analysis (always < 5% of the data set). According to Shapiro–Wilk test, data were normally distributed.

The mean RT values were analyzed by means of an analysis of variance (ANOVA) with OBJECT (cE vs. pE) and POSITION (Near vs. Far) as within subjects factor.

Newmann–Keuls post hoc analysis was used to interpret significant interactions. Values are presented as mean ± standard errors.

### TMS and Motor Imagery Experiment

TMS was used to evaluate changes in the left primary motor cortex excitability during MI and REST conditions, after 1 s from an acoustic cue. Further details about the timing of stimulations will be provided below in the *Experimental design* paragraph. Intensities were expressed as a percentage of the maximum output of the stimulator. TMS was performed with a single Magstim 200^2^ magnetic stimulator (Magstim Company) connected with a figure-of-eight coil with wing diameters of 70 mm. The coil was placed tangentially to the scalp with the handle pointing backward and laterally at a 45° angle to the sagittal plane inducing a posteroanterior current in the brain. This orientation was chosen based on the findings that the lowest motor threshold is achieved when the induced electrical current flows approximately perpendicular to the line of the central sulcus (Werhahn et al. 1994).

The coil was placed in order to evoke good responses both in the right APB and ECR muscles. Prior to the experimental procedure, the intensity of stimulation was individually defined to reliably elicit peak-to-peak motor-evoked potentials (MEPs) amplitude of a minimum of 1 mV in ECR muscle at rest. Since distal muscles are usually easier to stimulate, APB responses were higher than those of ECR. MEP size was calculated by averaging the amplitude of 20 trials recorded for each condition.

### EMG Recording

MEPs were recorded using silver disc surface electrodes taped to the belly and tendon of the muscles. The ground electrode was placed at the elbow. MEPs were recorded from right APB and ECR muscles using silver disc surface electrodes taped to the belly and tendon of the muscles. The ground electrode was placed at the elbow. Electromyographic signals (EMG) were digitalized, amplified and filtered (20 Hz to 1 kHz) with a 1902 isolated preamplifier controlled by the Power 1401 acquisition interface (Cambridge Electronic Design Limited, Cambridge, UK), and stored on a personal computer for display and later offline data analysis. Each recording epoch lasted 400 ms, of which 100 ms preceded the TMS.

### Experimental Design

Experiment consisted of 6 sessions performed randomly during the same day, for a total duration of about 2 h (Fig. 1—TMS and Motor Imagery experiment): BASELINE session, MI no-tool session, REST sessions while handling cE and pE, MI tools sessions while handling cE and pE.

During some trials, participants were instructed to perform a motor imagery task of a gesture related to fencing after the GO signal was provided. Specifically, they had to kinesthetically imagine (namely, imaging the sensation you feel when you perform a specific task) to perform a parry IV—attack combo. Sound cue was produced with a customizable microcontroller board (Arduino, Italy).

In particular, primary motor cortex excitability was tested in:

- **BASELINE session.** Participants were instructed to relax as much as possible and keep their eyes close. No mental task was required. TMS was delivered 1 s after the GO signal. (Fig. 1A).
- **MI no-tool session.** Subjects seated with their eyes closed without any tools. After the GO signal they had to kinesthetically imagine the fencing gestures. TMS was delivered 1 s after the GO signal, during the imagination task. At the end of each MI task, the experimenter asked the athlete whether the TMS was delivered while she/he was still involved in the imagery task. Trials acquired after the end of the imagery period were discarded and repeated (Fig. 1B).
- **REST sessions.** Subjects seated with their eyes closed, handling and lifting either cE or pE in a natural position (REST_cE_, REST_pE_). No mental task was required in these sessions. TMS was delivered 1 s after the GO signal (Fig. 1C).
- **MI tools sessions:** Subjects seated with their eyes closed handling and lifting either cE or pE in a natural position (MI_cE_; MI_pE_). Subjects were instructed to wait until the GO signal, and then to kinesthetically imagine the parry—attack. TMS was delivered 1 s after the GO signal. At the end of each MI task, the experimenter asked the athlete whether the TMS was delivered while she/he was still involved in the imagery task. Trials acquired after the end of the imagery period were discarded and repeated (Fig. 1D).

During REST sessions and MI tools sessions, subjects held the tools. To have a comparable facilitation along the experiment, an estimate of maximum voluntary isometric contraction (MVIC) was obtained from APB and ECR muscles with both objects before the TMS measurement. Participants learnt to hold the épées under the 10% of their MVIC. Trials with background EMG activity higher than 10% of MVIC were excluded from analysis.

Participants’ general motor imagery ability was evaluated by means of the Italian version of the Movement Imagery Questionnaire (MIQ-R). The MIQ-R is an 8-item self-report questionnaire, in which participants rated the vividness of their mental representations using 2 7-point scales, associated to kinesthetic and visual imagery: the score “1” means “really easy to feel/see”, whereas the score “7” corresponds to “really difficult to feel/see” (best score = 8, worst score = 56). On average, participants showed a motor imagery ability evaluated with MIQ-R of 20.83 ± 2.00 (SE).

### Data Processing and Statistical Analysis

MEP amplitude was measured peak-to-peak. According to Shapiro–Wilk test, data were normally distributed.

A paired *t*-test was adopted to compare MEPs recorded during MI no-tool session with respect to BASELINE, separately for ECR and APB muscles, to verify the effect of MI over the cortical excitability without any tool.

Trials requiring handling a tool with background EMG activity higher than 10% of MVIC were excluded from analysis. In all, 74 out of 3120 trials were discarded (2.37% of the total), and the statistical analysis was performed on the remaining trials. To exclude that the motor activity due to the handling of the different tools could influence the following MEPs, background EMG values 200 ms before to the stimulation were analyzed with a repeated measure ANOVA with OBJECT (cE vs. pE) and CONDITION (REST, MI) as within subjects factor. This analysis was repeated separately for ECR muscle and APB muscle.

Other data were analyzed with a repeated measure ANOVA with OBJECT (cE vs. pE) and CONDITION (REST, MI) as within subjects factor, in order to verify the specific effect of the tool over cortical excitability during MI. This analysis was repeated separately for ECR muscle and APB muscle with the same factors.

Newmann–Keuls post hoc analysis was used to interpret significant interactions.

Following results of the multisensory integration paradigm, we calculated the difference between the Far and the Near reaction time when subjects held the personal épée (ΔpE). To verify the influence of tool integration over the modulation of cortical excitability during MI, when ANOVA showed a significant OBJECT^*^CONDITION interaction, an analysis of covariance (ANCOVA) with ΔpE, an index of PPS variation, as covariate was run.

Newmann–Keuls post hoc analysis was used to interpret significant interactions. Values are presented as mean ± standard errors.

To further explore the relation between the integration of pE into PPS and the increment of the cortical excitability during MI with cE and pE, Spearman correlation was applied on ΔpE and changes in cortical excitability during MI, evaluated with the following formula: MI_pE_/REST_pE_ − MI_cE_/REST_cE_.

## Results

### Multisensory Integration Experiment

**Figure 2:**
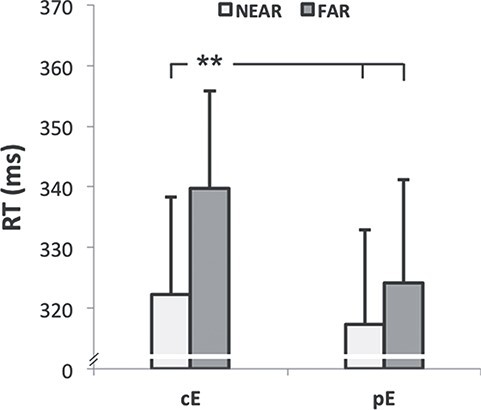
Averaged reaction times (RT, ms) recorded in Near and Far conditions in common épée (cE) and personal épée (pE) sessions. The error bars refer to the standard error of the mean. ^*^^*^refers to P < 0.01.

ANOVA showed an effect of the factor POSITION (*F_(1,12)_ = 12.25, P = 0.004*): the reaction times associated to Far audio stimuli (RT_Far_ *= 331.96 ± 11.53 m*s) were significantly higher than reaction times related to Near stimuli (RT_Near_ *= 319.77 ± 10.96* ms). Furthermore, a significant interaction OBJECT^*^POSITION was found (*F_(1,12)_ = 7.62, P = 0.017*). Post hoc test showed that, when subjects handled cE, their reaction times in the far position were significantly higher with respect to other conditions (cE RT_Far_ vs. cE RT_Near_: *339.71 ± 16.03* ms *vs. 322.29 ± 15.95* ms*, P = 0.000;* vs. pE RT_Near_: *317.24 ± 15.65* ms*, P = 0.000;* vs. pE RT_Far_: *324.21 ± 16.94* ms*, P = 0.000*), while there were no significant differences between reaction times in other conditions (*P > 0.05*). In particular, there was no significant difference between RT_Near_ and RT_Far_ when subjects handled pE (pE RT_Near_ vs. pE RT_Far_: *317.24 ± 15.65 vs. 324.21 ± 16.94 ms, P = 0.093 ms*) (Fig. 2).

### TMS and Motor Imagery Experiment

The results of the paired *t*-tests comparing MEP recorded during the BASELINE session and during the MI no-tool session showed that when fencers imagined the fencing gestures, despite a small increase in cortical excitability, the difference from baseline was not significant, neither in ECR (0.67 ± 0.09 mV vs. 0.88 ± 0.14 mV, *P = 0.254*) nor in APB (BASELINE vs. MI no-tool: 1.42 ± 0.23 mV vs. 1.72 ± 0.32 mV; *P = 0.340*) muscle.

ANOVA on EMG background activity for APB muscle shows no significant differences between the condition (*MIcE = 0.32 ± 0.05 mV; MIpE = 0.34 ± 0.05 mV; RESTcE = 0.31 ± 0.05 mV; RESTpE = 0.32 ± 0.06 mV; P always > 0.42)*, such as ANOVA on MVIC activity in ECR muscle (*MIcE = 0.27 ± 0.04 mV; MIpE = 0.25 ± 0.03 mV; RESTcE = 0.23 ± 0.03 mV; RESTpE = 026 ± 0.04 mV; P always > 0.17)*.

**Figure 3:**
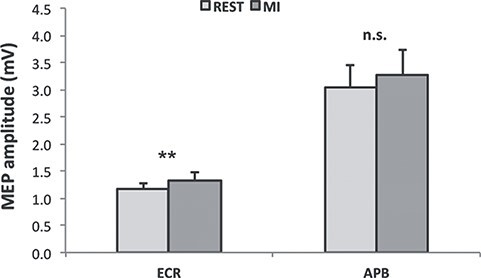
MEP amplitudes of averaged REST conditions (light gray) and MI tool conditions (dark gray), acquired from ECR muscle (left) and APB muscle (right). The error bars refer to the standard error of the mean. ^*^^*^refers to P < 0.01.

Concerning ECR muscle, ANOVA comparing REST sessions and MI tool sessions with both personal and common épée showed a significant effect of the CONDITION factor. Post hoc analysis revealed that MEP increased during imagination significantly (REST vs. MI: 1.16 ± 0.10 mV < 1.33 ± 0.14 mV*, P = 0.007*) (Fig. 3).

**Figure 4:**
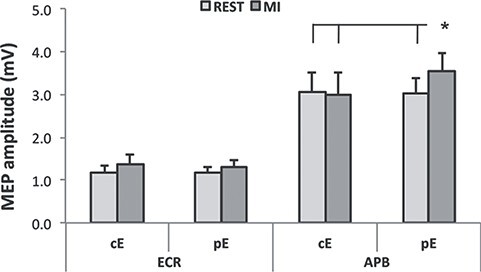
ANOVA results. MEP amplitudes acquired from ECR (left) and APB (right) while participants held their common épée (left) and personal épée (right) in “REST” sessions (light gray) and “MI-tools” sessions (dark gray). The error bars refer to the standard error of the mean. ^*^refers to P < 0.05.

ANOVA on APB comparing REST sessions and MI tool sessions with both common and personal épée showed a significant interaction between OBJECT and CONDITION (*F_(1,12)_ = 5.52, P = 0.037)*. Post hoc analysis revealed that during imagination while handling the personal tool cortical excitability increased significantly with respect to its REST condition (*MI_pE_ vs. REST_pE_: 3.54 ± 0.43 mV > 3.03 ± 0.36 mV, P = 0.032*). MI_pE_ was also significantly higher with respect to all the cE conditions (*MI_pE_ vs. REST_cE_: 3.54 ± 0.43 mV > 3.07 ± 0.43 mV; vs. MI_cE_: 3.00 ± 0.52 mV; P always < 0.03*). There was no significant difference between *REST_cE_* and *MI_cE_* (*P = 0.916*)*.* Notably, REST_pE_ did not differ significantly from REST_cE_ (*P = 0.830*) (Fig. 4).

Further, ANCOVA for APB muscle showed a significant interaction between OBJECT and POSITION *(F_(1,11)_ = 5.70, P = 0.036)*. Post hoc analysis confirmed that during MIpE the cortical excitability increased significantly with respect to its REST condition and with respect to both the cE conditions. To explore the impact of the variation of ΔpE, namely the parameter introduced as covariate in the statistical analysis, over the cortical excitability, we explore the β coefficients. A β coefficients of −0.21 was associated with MIpE showing that MEP amplitude during MI increased most when subjects have lower ΔpE, index of PPS variation.

**Figure 5:**
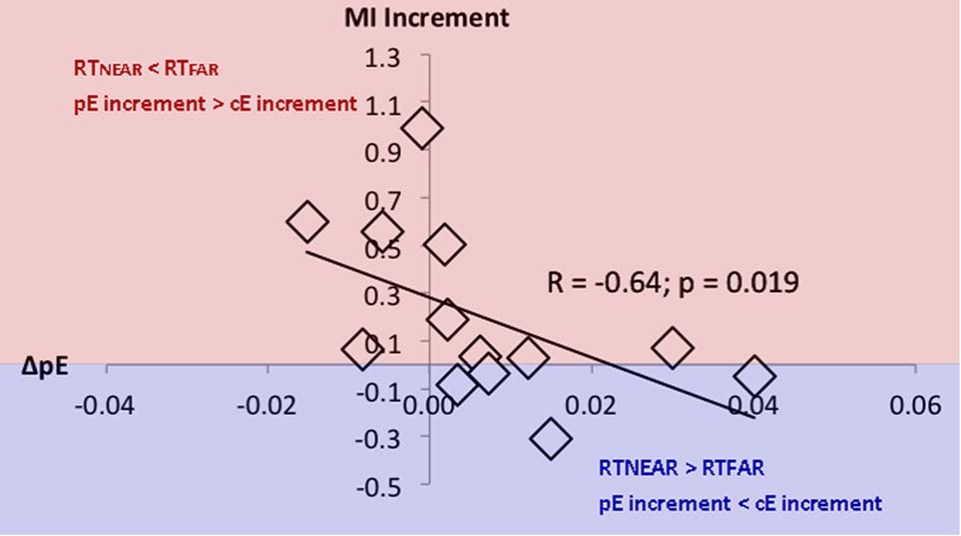
Spearman correlation between ΔpE, as an index of PPS variation, and MI increment, representing changes in cortical excitability during MI when handling each tool. The red area represents a higher increase of MI with respect to the REST when subjects handled personal épée (pE) with respect to the common épée (cE), the blue area represents the opposite.

At last, a significant negative relationship was found (*R* = −0.64; *P =* 0.0191); namely, the lower the ΔpE values, the greater the difference between the increasing of the MI with the 2 épée. (Fig. 5).

## Discussion

The aim of the present study was to assess whether the familiarity with a specific tool affects not only PPS extension, but also cortical motor representation in athletes. In particular, we tested in a group of elite fencers, through 2 different experiments, if the épée they use during training and a generic 1 were differently embodied and differently integrated in their motor representation. Data confirmed previous findings that only the personal épée stably enlarges the PPS of the athletes (Biggio et al. 2017). Furthermore, during an MI task, primary motor cortex excitability of a distal muscle, involved in holding the tool, only increased while subject handled the personal épée. On the contrary, proximal muscles’ cortical excitability increased regardless the handled épée. This finding suggests that cortical motor representation of the muscles involved in controlling the haptic contact with the tool is specifically modulated by the long-term experience with it.

Concerning PPS, it is known that its boundaries enlarges immediately after a few minutes of practice with an unfamiliar object, and lasts only for short time intervals, but this change does not occur when the object is only passively handled before the test, showing the strict connection between PPS and the motor components of external world interaction (Farnè et al. 2005). Different from this short-term expansion is a long-term PPS enlargement that is durable in time and does not expire after short time periods. This is strictly linked to the familiarity with the tool. For example, in blind subjects, a population using canes as a tool to explore the surrounding environment, it was shown that the PPS enlarged to embody the cane stably (Serino et al. 2007). In a following study, Bassolino et al. (2010) further explored the relation between expertise and PPS in a group of long-term experience mouse-user, confirming that passively holding a known tool triggers an extension of PPS. Furthermore, they explored the extension of the PPS of the nondominant hand, showing that long-term experience did not transfer to the other hand and suggesting that “expert users develop different representations of space through experience, that these representations co-exist, and that they can be dynamically and functionally engaged depending on contextual demands”.

Sport experience could constitute a representative model to investigate the influence of tool-use in space representation. Indeed, in a previous study, we found that tennis players stably integrated in their PPS the tennis racket, but only the specific 1 used during their daily training, showing that the effective functional properties of the tool are crucial in extending PPS (Biggio et al., 2017). This finding is in agreement with the result showed in this work and strongly suggests that every sport requiring the frequent use of the same tool could lead to an integration of that specific equipment, depending on the level of familiarity with it. It has been suggested that tool-use not only modifies our perception of the world around us (Baccarini et al. 2014), letting us to improve the anticipation of action possibilities with the tool (Bourgeois et al. 2014), but also it modifies the spatial metric of our own body (Cardinali, Frassinetti et al. 2009; Sposito et al. 2012; Canzoneri, Marzolla et al. 2013; Baccarini et al. 2014).

**Figure 6:**
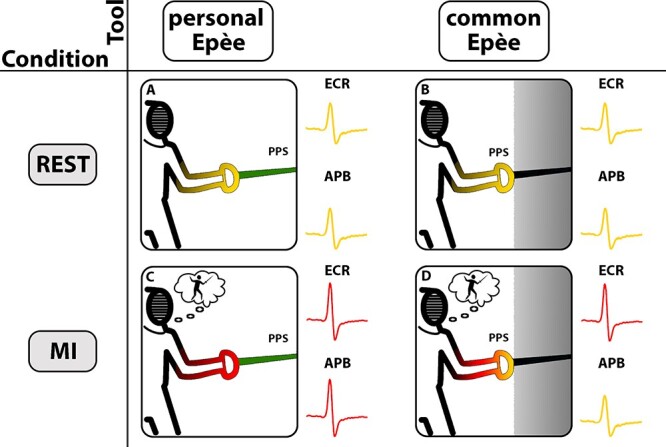
Results summary. Each box represents an experimental session. Box A represents REST pE (in green) session, Box B represents REST cE (in black) session, Box C represents MI pE (in green) session, Box D represents MI cE (in black) session. The yellow and red shades in false color scale over fencer’s arms represent the cortical motor excitability of the proximal and distal muscles. Nearby to each box the recorded MEPs are summarized for ECR and APB muscles, respectively. The yellow MEP represents the amplitude of MEP in the REST conditions, while the red MEP represents a significant increase of the MEP amplitude with respect to REST condition. The gray background represents the boundaries of PPS, which are modulated by tools.

In fact, in order to efficiently act in space, we need to keep the representation of our body updated for both current posture and limb position (Vargas et al. 2004; de Lange et al. 2006). This also includes the kinesthetic properties of the handled tool (Maravita and Iriki 2004). With reference to body representation, it has been proposed that 2 different aspects of tools use can influence its embodiment (Cardinali et al. 2016): a morpho-functional output, referring to the kind of action you can perform with a tool, and a sensorimotor properties, the action you have to perform with the tool (Weser and Proffitt 2019). Adopting this distinction, we suggest that sensorimotor properties of tools can influence not only the PPS but also the internal models of action which are modulated by the gained experience during both overt and covert training protocols. Therefore, we studied the cortical motor representation of athletes during an MI task to understand if tools that modulate differently the PPS are also differently integrated in the internal model of actions requiring their use. Toward this goal, fencers had to imagine a sport-related gesture that involved the use of a tool, with or without handling an épée. Notably, they did not show an increased motor cortex excitability during the mere MI, in line with previous literature. In fact, corticospinal excitability during MI with an object was modulated by actually touching an object, through the combination of tactile and proprioceptive inputs (Mizuguchi et al. 2011). Indeed, MI abilities have been consistently shown to vary as a function of the afferent inputs from the periphery. Skilled performance (e.g., racket sports) involves sensorimotor tasks, requiring a close coupling of actions with sensory inputs (Lees 2003). It has been shown that both visual and somatosensory information influences brain activity during motor imagery (Vargas et al. 2004; Fourkas et al. 2006; Mercier et al. 2008). Zhang et al. (2018) found in basketball players an increased temporal congruence between MI and motor execution, and a higher vividness of the imagined movement, but only when holding a ball. This is in agreement with a previous study of our group showing that the isochrony between MI and movement execution in expert tennis players was maintained only when athletes handled the tennis racket (Bisio et al. 2014).

Further, we studied cortical excitability from 2 different muscles, a proximal one, involved in the strength component of the defensive and attack actions, and a distal one, controlling the haptic contact with the tool. In particular, subjects had to hold a common épée and their own personal épée used during training. Results showed a different modulation induced by common and personal épée on ECR and APB muscles, respectively. ECR’s MEPs in REST conditions (Fig. 6A, B) were significantly lower with respect to MEPs recorded when subjects handled a sport-related tool during MI (Fig. 6C, D). On the contrary, only the personal tool was able to evoke a greater response in APB muscle during MI with respect to REST condition (Fig. 6 D).

Fencers were required to perform a very specific MI task, namely imaging to execute a series of movement that they are trained to do with a specific weapon in the most automatic, rapid and effective way. It can be suggested that a reliable process of motor imagery might initiate in the case of match between the afferent information and those included in the athletes’ cortical motor representation, which are specific for the sport gesture. Moreover, the afferent information regarding the physical properties of a tool (new or familiar) could be useful in an early phase, during the retrieving of the correct motor representation. People are able to imagine and also execute a well-known action with a brand new object. This can also be useful in learning how to use other tools. However, the process to distinguish between 2 épées that have the same features should be very accurate. Even if fencers usually wear gloves, the body part that is always in contact with the tool is the hand. One might speculate that afferent inputs sending information about the properties of familiar tool are sensed specifically from distal muscles. It is known that the increase of corticospinal excitability is specific to the muscle involved in the imagined movement. For instance, an increase of MEP amplitude in the biceps brachii muscle was observed during MI of elbow flexion but not during MI of elbow extension, and inversely for the triceps brachii muscle (Fadiga et al. 1999). Regarding fine movements involving distal muscles, MI of index abduction increased MEP of first dorsal interosseus but not MEP of abductor digiti minimi neither MEP in forearm muscles such as ECR (Rossini 1999). In the case of the common tool 1 could hypothesize that APB was not involved in the MI task, because without knowing the properties of the tool the gesture is not generalized in athletes’ motor plan. It may be assumed that handling an object, different from the 1 already included in motor plans, weakens the motor representation of the muscles that are in contact with it. This mechanism could be similar to what happens when imagining a tool-movement without touching any.

It is worth noting that when subjects just held both the épées without performing a mental task no differences appeared in MEP’s amplitude between REST conditions. This suggests that the differences between tools emerged only with action-oriented goal, as occurred during motor imagery or while detecting stimuli in multisensory integration paradigm. Other motor-related mental processes show a similar muscular specificity. During action observation, the motor resonance, namely the activation of the observer motor system, was found on the specific muscle involved in gesture (Bisio et al. 2015). Nonetheless, muscular specificity is modulated by contextual features influencing the observer (Lagravinese et al. 2017).

Our result further showed a strict interaction between the long-term integration of a tool inside the PPS of the subjects and the excitability of motor system during MI. Specifically, the lower difference between RT in Far and Near position explains a major increase of cortical excitability of APB muscle during MI with the personal épée compared to REST condition. Therefore, a stable integration of the tool in PPS seems to correspond with the inclusion of it in the action internal models of the athletes.

The causal link between these 2 aspects is not yet known. It is known that even a brief use of a tool could induce a short-term enlargement of the PPS that expires shortly after. Conversely, a long-term enlargement occurs for more familiar tools. To our knowledge, up to now, it has not been explored when a tool becomes so familiar to trigger a long-term integration in the PPS. Further, it is not easy to determine when the internal model of an action has been consolidated and becomes a real expertise of a subject. When using a tool, these 2 aspects could develop together, but it could also be the case that the long-term integration of a tool inside the body representation can influence the establishment of internal models of actions integrating the specific tool. Further studies are needed to explore the casual link between these 2 aspects.

In conclusion, we can assume that the familiarity with tools during motor tasks does not only influence the activity of the sensory and associative cortical areas, modifying the space representation, but also the cortical motor representation of those muscles involved in the control of the haptic contact. In our opinion, this notion could not be restricted to sport but it can be applied to other fields of study, such as rehabilitation, when the use of prosthetic aids is suggested to be used by the patients.

## Notes

We thank Dr Alessio Signori for the help with statistical analysis. *Conflict of Interest*: None declared.
